# Lactitol Alleviates Loperamide-Induced Constipation in Sprague Dawley Rats by Regulating Serotonin, Short-Chain Fatty Acids, and Gut Microbiota

**DOI:** 10.3390/foods13132128

**Published:** 2024-07-03

**Authors:** Joo Hyun Jang, Sang Min Kim, Hyung Joo Suh, Minchul Gim, Hoyeon Shin, Hyunsook Jang, Hyeon-Son Choi, Sung Hee Han, Yeok Boo Chang

**Affiliations:** 1 Department of Integrated Biomedical and Life Science, Graduate School, Korea University, Seoul 02841, Republic of Korea; actionplan@ckdhc.com (J.H.J.); sangmin_i@naver.com (S.M.K.); suh1960@korea.ac.kr (H.J.S.); 2 Transdisciplinary Major in Learning Health Systems, Department of Healthcare Sciences, Graduate School, Korea University, Seoul 02841, Republic of Korea; 3 LOTTE R&D Center, Seoul 07594, Republic of Korea; minchul.gim@lotte.net (M.G.); hoyeon.shin@lotte.net (H.S.); hyunsook.jang@lotte.net (H.J.); 4 Department of Food and Nutrition, Sangmyung University, Seoul 03016, Republic of Korea; choice120@gmail.com; 5 Institute of Human Behavior and Genetics, Korea University, Seoul 02841, Republic of Korea; sungheeh@korea.ac.kr

**Keywords:** lactitol, constipation, animals, gut microbiota, loperamide, gastrointestinal transit

## Abstract

The objective of this study was to examine the impact of lactitol on constipation caused by loperamide in Sprague Dawley rats, with a particular emphasis on its underlying mechanisms and potential health advantages. The lactitol effectively improved fecal parameters, intestinal tissue structure, and the expression of constipation-related gene expression and proteins. Lactitol alleviated fecal weight and water content altered by loperamide and enhanced gastrointestinal transit. The administration also restored mucosal and muscular layer thickness. Mechanistically, lactitol upregulated the mRNA expression and/or protein levels of mucins (MUC2 and MUC4), occludin, claudin-1, and zonula occludens, indicating improved intestinal barrier function. Lactitol positively regulated the composition of cecal microbiota, leading to an increased relative abundance of *Bifidobacterium*, *Lactobacillus*, and *Romboutsia*. Conversely, lactitol decreased the relative abundance of *Prevotella*, *Aerococcus*, *Muribaculum*, *Blautia*, and *Ruminococcus*. This study demonstrated the potential of lactitol to relieve constipation by modulating the gut microbiota. These findings suggest that lactitol is an alternative to traditional laxatives and has potential as a health-promoting food sweetener.

## 1. Introduction

In modern society, digestive discomfort is a common health issue affecting people across all age groups. Many individuals suffer from digestive symptoms such as bloating, gas, indigestion, and constipation, which reduce their quality of life by limiting daily activities. Discomfort in the digestive system is caused by various factors, including stress/anxiety, irregular meals, lack of fiber and water intake, and lack of physical activity [1]. Constipation, a representative digestive discomfort, is a widespread health concern characterized by infrequent bowel movement, difficulty passing stools, a sense of incomplete evacuation, reduced defecation (less than three times a week), and a feeling of pain during defecation [2]. These constipation symptoms are reported to occur more frequently in women and the elderly [3].

Constipation medications have evolved over time, starting from herbal and dietary remedies, such as senna and aloe vera used in ancient Egypt. In the 20th century, synthetic laxatives like bisacodyl, polyethylene glycol, and lactulose were developed. In recent decades, novel classes of laxatives have emerged, including bulk-forming agents and osmotic agents [4]. Bulk-forming laxatives absorb water and form soft, bulky stools that stimulate regular bowel movements. Indigestible fiber, a type of bulk-forming laxative, improves constipation by absorbing water in the large intestine, softening the stool, increasing stool volume, and facilitating excretion [5]. The consumption of dietary fiber from sources, such as brown rice, psyllium seeds, and methyl cellulose derivatives, is safe and has few side effects [6].

Osmotic laxatives, categorized as saline and hyperosmotic laxatives, facilitate bowel movement by drawing moisture into the intestines. Typical saline laxatives, including magnesium, sulfate, phosphate, and citrate, are commonly prescribed drugs that draw water into the intestine through osmosis, softening the stool, and stimulating bowel movements [6]. However, saline laxatives can lead to electrolyte imbalances [7], which are responsible for symptoms such as muscle weakness, cramps, and cardiac arrhythmias. Additionally, their long-term use can cause dehydration, diarrhea, and excessive discomfort. Hyperosmotic laxatives comprise sugar alcohols or sugar substitute derivatives, such as lactulose, lactitol, sorbitol, glycerin, and polyethylene glycol [8]. These non-absorbable polysaccharide laxatives are utilized by bacteria in the large intestine, increasing intestinal osmotic pressure and acidity. Consequently, moisture is accumulated, resulting in improved stool softening and increased defecation frequency [9]. Non-absorbable polysaccharide laxatives are often less preferred by individuals due to their sweet taste, potentially resulting in discomfort such as abdominal distension and flatulence caused by gas generated during metabolism. Nevertheless, the utility of these laxatives is promising, with few side effects, and they are particularly beneficial for specific groups, including the elderly, pregnant women, those with diabetes, and individuals with compromised liver function. Further, they can be safely prescribed for a long period even in patients with decreased or impaired kidney function [10].

Lactitol (β-galactoside-sorbitol), a lactose-derived sugar alcohol and a disaccharide analog of lactulose, serves as a low-calorie sweetener and is recognized for its laxative effects. Lactitol also acts as a prebiotic, promoting beneficial intestinal microorganisms, such as *Bifidobacterium* and *Lactobacillus* while inhibiting pathogens [11,12]. Several studies have demonstrated the laxative effects of lactitol [10,13]. Clinical research indicates that lactitol effectively alleviates the symptoms of adult constipation by increasing stool frequency [13]. A meta-analysis showed that lactitol is preferred over lactulose, another osmotic laxative, for treating chronic constipation due to its superior efficacy, better palatability, and lower incidence of adverse events [14]. However, comprehensive investigations into its impact on constipation, including molecular mechanisms and microbiota analysis, remain limited.

This study confirmed the effect of lactitol on a loperamide-induced constipation animal model by analyzing fecal parameters, intestinal biomarkers related to constipation, and microbiome composition. The findings provide a comprehensive understanding of lactitol-mediated improvements in constipation and suggest its potential use as a promising edible laxative.

## 2. Materials and Methods

### 2.1. Chemicals

Loperamide was purchased from Sigma-Aldrich (St. Louis, MO, USA), and lactulose was obtained from JW Pharmaceutical Co., Ltd. (Seoul, Republic of Korea). Lactitol was provided by Danisco, Inc. (Thomson, IL, USA). β-actin, aquaporin 3 (AQP3), c-kit, and anti-rabbit IgG were obtained from Cell Signaling Technology (Danvers, MA, USA). Zonula occludens-1 (ZO-1) was purchased from Santa Cruz Biotechnology (Dallas, TX, USA).

### 2.2. Animals and Experimental Design

The animal study protocol was approved by the Korea University Institutional Animal Care and Use Committee (KUIACUC-2021–0100), and the animal experiments were approved by the Korea University Animal Intuitive Care Committee (KUIACUC-2023-0028). Male Sprague Dawley (SD) rats (160–180 g, 6 weeks old) were purchased from Orient Bio (Seongnam, Republic of Korea). Drinking water and feed were freely provided in a breeding room with a temperature of 22 ± 1 °C, a relative humidity of 63 ± 5%, and a 12 h light/dark cycle. After an adaptation period of one week, to induce constipation, loperamide (5 mg/kg) was administered orally twice a day for one week to all groups, except for the normal group. Thereafter, each group received orally administered loperamide once daily for four weeks. The experimental groups were divided into a total of six groups (eight rats/group) as follows: normal (NOR, no loperamide), control (CON, loperamide control group, 5 mg/kg), positive control (PC, lactulose 2000 mg/kg with loperamide), low-dose lactitol administration (LTL, lactitol 300 mg/kg with loperamide), medium-dose lactitol administration (LTM, lactitol 500 mg/kg with loperamide), and high-dose lactitol administration (LTH, lactitol 800 mg/kg with loperamide).

### 2.3. Analysis of Fecal Parameters

During the sample administration period, stool samples were collected once a week from the third week, and the fecal number, weight, and water content were measured. Fecal water content was calculated as the weight difference before and after drying the feces at 70 °C for 24 h [15].

### 2.4. Measurement of Intestinal Transit Rate

A day before sacrifice, SD rats were fasted for 18 h, and changes in the digestive tract migration rate due to sample administration were measured using charcoal [16]. Charcoal solution (1 mL; 5% charcoal in 0.5% carboxymethyl cellulose solution) was administered orally, and the rats were sacrificed 30 min later. Total intestinal length and charcoal migration distance were measured, and the digestive tract migration rate was calculated.

### 2.5. Histological Analysis

For hematoxylin-eosin (H&E) staining of the intestinal tissue, paraffin-embedded tissue sections were deparaffinized with xylene, hydrolyzed for 5 min in 70–100% ethanol, and stained in hematoxylin solution (Sigma-Aldrich) for 3 min [17]. The stained tissue was washed with water and stained again for 3 min with the eosin solution. Following staining, the tissue was washed thoroughly in distilled water, dehydrated for 5 min in 70–100% ethanol, washed with xylene, and sealed.

For Alcian blue staining, crypt cells in the colon were deparaffinized in the same way as for H&E staining, treated with ethanol, and then stained (pH 2.5) for 30 min. The stained tissue was washed with water and stained again for 5 min using a nuclear fast-red solution. After the dyeing was completed, it was washed in running water, dehydrated for 5 min in 70–100% ethanol, washed with xylene, and sealed.

Immunohistochemical (IHC) staining to observe intestinal interstitial cells of Cajal (ICCs) was performed in the same way as H&E staining by deparaffinizing, treating with ethanol, and then staining with a c-kit primary antibody (SC-168, Santa Cruz Biotechnology). After the dyeing was completed, it was washed in distilled water, dehydrated for 5 min in 70–100% ethanol, washed with xylene, and sealed. The stained intestinal mucosal cells were observed under an optical microscope (ZEISS, Axiovert S100, Jena, Germany) [18].

### 2.6. Analysis of Serotonin and Substance P Content in Colon Tissue

Serotonin and substance P levels in the intestinal tissue were analyzed using a serotonin ELISA kit (MBS1601042, MyBioSource, San Diego, CA, USA) and substance P kit (MBS702782, MyBioSource), respectively. After adding 1 mL of phosphate-buffered saline to 200 mg of colon tissue, the tissue was homogenized and centrifuged (8000× *g*, 20 min, 5 °C). Thereafter, the supernatant was recovered and analyzed according to each manufacturer’s instructions.

### 2.7. Gene Expression Level Analysis by qPCR

Total RNA was isolated from 100 mg of intestinal tissue using TRI reagent (Thermo Fisher Scientific, Rockford, IL, USA) [19]. Briefly, total RNA (1 μg) was reverse-transcribed into cDNA using a cDNA kit (Thermo Fisher Scientific) with 50 µM oligo-(dT) primer and 10 mM dNTP mix. RNase H (Thermo Fisher Scientific) was added to remove RNA at 37 °C for 20 min. For PCR, gene-specific primers, SYBR green gene expression master mix (Applied Biosystems, Waltham, MA, USA), and 1 µL of synthesized cDNA diluted with DEPC water were used. Fifty cycles were performed at 50 °C for 2 min for denaturation, 10 min at 90 °C for annealing, and 1 min at 60 °C for extension. The expression of each target primer gene was determined relative to that in the normal. Primers used in the experiment are listed in Table S1.

### 2.8. Western Blot Analysis

For Western blotting, 100 mg of intestinal tissue was homogenized in lysis buffer, and the supernatant was collected by centrifugation (12,000 rpm, 5 min, 4 °C). Protein concentration was determined using the BCA protein assay kit (Thermo Fisher Scientific, Rockford, IL, USA). Thirty micrograms of protein were separated by 12% SDS-PAGE and transferred to a PVDF membrane. The membrane was blocked with 5% BSA for 1 h, then incubated with primary antibody (anti-rabbit IgG, Cell Signaling Technology) at 4 °C for 2 h. After washing with TBST, the secondary antibody was added and incubated at room temperature for 2 h. Detection was performed using SuperSignal™ Western Blot Enhancer and visualized with the FluorChem M Fluorescent Western Imaging System. The antibodies used included β-actin, ZO-1, C-kit, MUC4, and AQP3 (all from Cell Signaling Technology).

### 2.9. Short Chain Fatty Acid (SCFA) Analysis

The ceca of the SD rats were removed, and methanol extracts were prepared and analyzed for SCFA content using gas chromatography (GC, Agilent Technologies, Santa Clara, CA, USA) with a DB-FFAP column (50 m × 0.32 mm × 0.50 μm). The column temperature was maintained at 100 °C for 1 min, raised to 180 °C at a rate of 8 °C/min, up to 200 °C at a rate of 20 °C/min, and then maintained at 200 °C for 5 min. SCFA content in the cecum was analyzed using acetic, propionic, butyric, and valeric acids as standard.

### 2.10. Cecal Microbiota Analysis

Cecal microbiota analysis was conducted on six SD rats per group. DNA extraction from 100–250 mg of cecum was performed using the QIAamp PowerFecal Pro DNA Kit (Qiagen, Hilden, Germany). DNA concentration was adjusted to 5 ng/µL. Two-step PCR with 341F and 806R primers amplified the V3–4 region of the 16S rRNA gene. This was implemented using Illumina MiSeq library [20] and performed according to the manufacturer’s protocol. Sequencing was undertaken by Macrogen (Seoul, Republic of Korea).

Data analysis of the changes in the intestinal microbial community was performed using a bioinformatics tool. Using the CD-HIT-OTU program, OTUs were generated by removing noise data considered sequencing errors and performing clustering using a sequence similarity cutoff (97%). The QIIME program was used to perform taxonomic assignments and diversity statistics [21]. To analyze community diversity within the sample, alpha diversity was analyzed using the Chao1, Shannon, and Inverse Simpson indices. In addition, beta-diversity analysis for community diversity between samples was performed using principal coordinate analysis (PCoA) with the weighted UniFrac distance analysis method.

### 2.11. Statistical Analysis

Data were expressed as mean ± S.E.M. Statistical analysis was conducted using SPSS (v22.0; IBM, NY, USA). One-way ANOVA and Tukey’s multiple test (post hoc) were used to assess differences, with significance set at *p* < 0.05.

## 3. Results

### 3.1. Effect of Lactitol on Fecal Parameters in SD Rats with Loperamide-Induced Constipation

**Figure 1:**
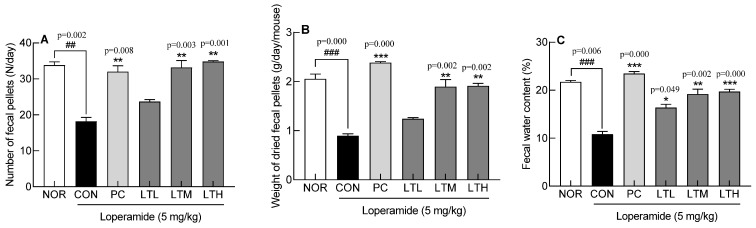
Effect of lactitol on (**A**) the number of fecal pellets, (**B**) weight of dried fecal pellets, and (**C**) fecal water contents in SD rats treated with loperamide. NOR: normal group; CON: loperamide-control group (5 mg/kg), PC: lactulose (2010 mg/kg) treated with loperamide; LTL: low dose of lactitol (300 mg/kg) treated with loperamide; LTM: medium dose of lactitol (500 mg/kg) treated with loperamide; LTH: high dose of lactitol (800 mg/kg) treated with loperamide. ^##^ *p* < 0.01 and ^###^ *p* < 0.001 vs. NOR, and * *p* < 0.05, ** *p* < 0.01, *** *p* < 0.001 vs. CON.

Fecal parameters were measured to evaluate the effect of lactitol treatment on constipation in SD rats induced by loperamide (Figure 1). The CON showed significantly lower fecal number, weight, and water content than the NOR (*p* < 0.01, *p* < 0.001, and *p* < 0.001, respectively). The PC, LTM, and LTH had significantly higher fecal numbers than the CON (*p* < 0.01). The fecal dry weights in the PC, LTM, and LTH were notably higher than those in the CON (*p* < 0.001, *p* < 0.01, and *p* < 0.01, respectively). The fecal water content also significantly recovered in the PC and lactitol treatment groups (Figure 1C, *p* < 0.001, *p* < 0.05, *p* < 0.01, and *p* < 0.001, respectively). Fecal parameters that were reduced by loperamide treatment were recovered by medium and high doses of lactitol.

### 3.2. Effect of Lactitol on Gastrointestinal Transit Rate in SD Rats with Loperamide-Induced Constipation

**Figure 2:**
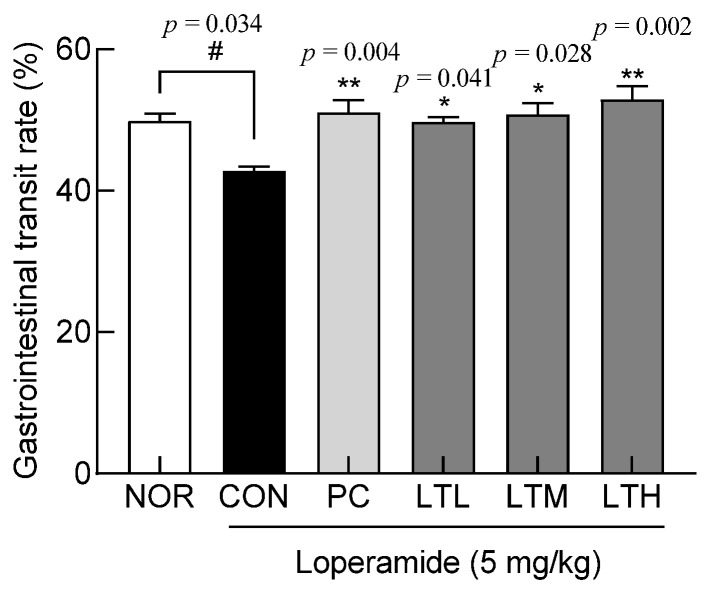
Effect of lactitol on gastrointestinal transit rate in SD rats treated with loperamide. NOR: normal group; CON: loperamide-control group (5 mg/kg), PC: lactulose (2010 mg/kg) treated with loperamide; LTL: low dose of lactitol (300 mg/kg) treated with loperamide; LTM: medium dose of lactitol (500 mg/kg) treated with loperamide; LTH: high dose of lactitol (800 mg/kg) treated with loperamide. ^#^ *p* < 0.05 vs. NOR, and * *p* < 0.05 and ** *p* < 0.01 vs. CON.

To examine the effect of lactitol on improving intestinal function, the digestive tract transit rate was measured by assessing the intestinal transit of charcoal (Figure 2). The gastrointestinal transit rate of the NOR administered only drinking water was 49.9%, and that of the CON administered only loperamide was 42.7%, indicating a significant difference between the two groups (*p* < 0.05). Intestinal transit rates in the LTL, LTM, and LTH were significantly higher than those in the CON (*p* < 0.05, *p* < 0.05, and *p* < 0.01, respectively). The PC showed a gastrointestinal transit rate of 51.8%. As the lactitol treatment dose increased, the intestinal transit rate of charcoal significantly increased compared with that in the CON.

### 3.3. Intestinal Epithelial Cells through H&E Staining

**Figure 3:**
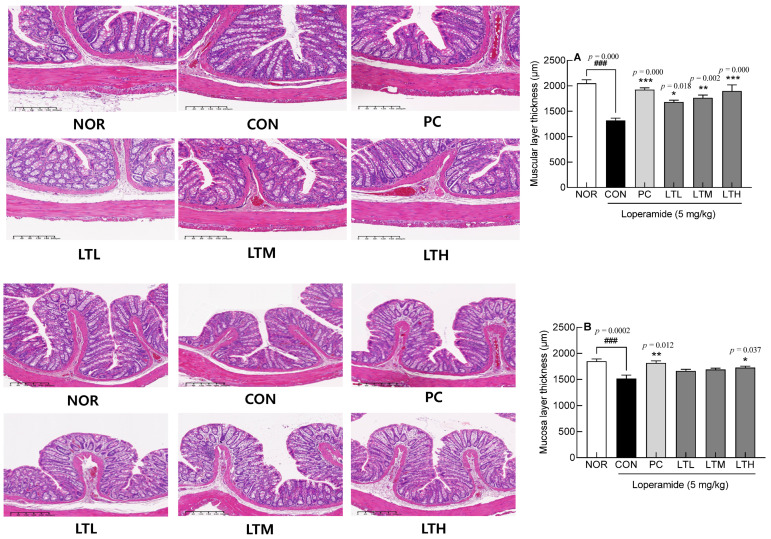
Effect of lactitol on (**A**) muscular layer and (**B**) mucosal layer thickness in SD rats treated with loperamide. NOR: normal group; CON: loperamide-control group (5 mg/kg), PC: lactulose (2010 mg/kg) treated with loperamide; LTL: low dose of lactitol (300 mg/kg) treated with loperamide; LTM: medium dose of lactitol (500 mg/kg) treated with loperamide; LTH: high dose of lactitol (800 mg/kg) treated with loperamide. ^###^ *p* < 0.001 vs. NOR, and * *p* < 0.05, ** *p* < 0.01 and *** *p* < 0.001 vs. CON.

The thickness of the mucosal and muscular layers of the intestinal epithelial cells related to the movement of colonic contents was observed using H&E staining (Figure 3). The thickness of the muscle layer of the intestinal epithelial cells was significantly lower in the CON than that in the NOR (Figure 3A, *p* < 0.001). In the lactitol-treated groups, the thickness recovered significantly in proportion to the lactitol concentration (Figure 3A; *p* < 0.05, *p* < 0.01, and *p* < 0.001, respectively).

The thickness of the mucosal layer of intestinal epithelial cells in the CON was significantly lower than that in the NOR (Figure 3B, *p* < 0.001). Lactitol administration significantly restored the thickness of intestinal epithelial cells in proportion to the concentration (Figure 3B, *p* < 0.05, *p* < 0.01, and *p* < 0.01, respectively). The thickness of the mucosal and muscle layers of epithelial cells was similar between the PC and LTH, with no significant variations observed. These results suggest that lactitol treatment improved the thickness of the mucosal and muscle layers of intestinal epithelial cells, which was reduced by constipation.

### 3.4. Effect of Lactitol on Intestinal Crypt Cells and Interstitial ICCs

**Figure 4:**
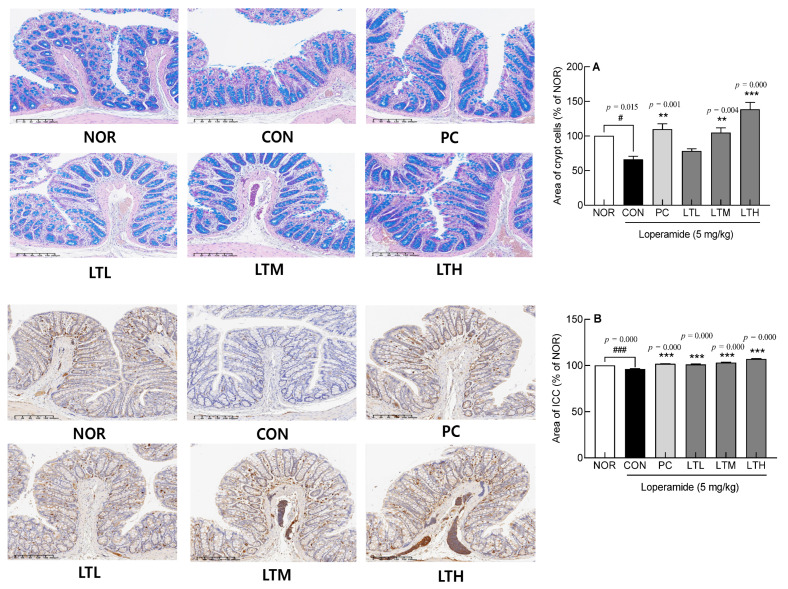
Effect of lactitol on (**A**) crypt cells and (**B**) ICC distribution in SD rats treated with loperamide. NOR: normal group; CON: loperamide-control group (5 mg/kg), PC: lactulose (2010 mg/kg) treated with loperamide; LTL: low dose of lactitol (300 mg/kg) treated with loperamide; LTM: medium dose of lactitol (500 mg/kg) treated with loperamide; LTH: high dose of lactitol (800 mg/kg) treated with loperamide. ^#^ *p* < 0.05 and ^###^ *p* < 0.001 vs. NOR, and ** *p* < 0.01 and *** *p* < 0.001 vs. CON.

The distribution of crypt cells in the CON, in which constipation was induced by loperamide, was significantly lower than that in the NOR (Figure 4A, *p* < 0.05). The administration of lactitol resulted in a dose-dependent increase in crypt cell distribution. Rats in the LTM and LTH exhibited significantly greater levels of crypt cell distribution compared to the CON (Figure 4A, *p* < 0.01, and *p* < 0.001, respectively). Lactulose treatment (PC) also resulted in a significantly higher crypt cell distribution than that in CON, and there was no significant difference in crypt cell distribution between the PC and LTM.

The distribution of ICC involved in intestinal peristalsis was observed using IHC staining. The CON showed a significantly lower ICC distribution than the NOR (Figure 4B). Lactitol administration significantly increased the ICC distribution in proportion to the concentration (Figure 4B, *p* < 0.001). The PC exhibited a significantly greater distribution of ICCs compared to the CON, while no notable difference was detected between the LTL and LTM. Thus, treatment with lactitol can help expel colonic contents by increasing the distribution of crypt cells involved in intestinal mucosal secretion and ICCs, which are involved in intestinal motility.

### 3.5. Effects of Lactitol on Serotonin and Substance P Content in Colon Tissue of SD rats with Loperamide-Induced Constipation

**Figure 5:**
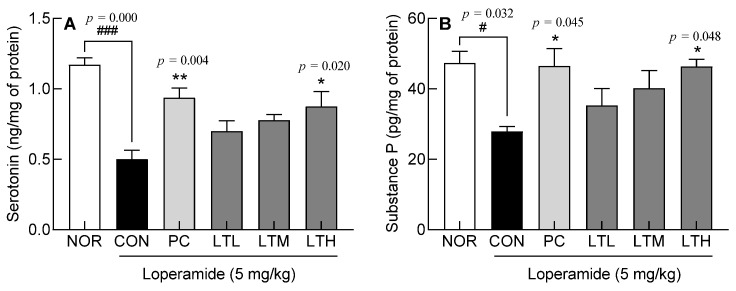
Effect of lactitol on serotonin (**A**) and substance P (**B**) content in SD rats treated with loperamide. NOR: normal group; CON: loperamide-control group (5 mg/kg), PC: lactulose (2010 mg/kg) treated with loperamide; LTL: low dose of lactitol (300 mg/kg) treated with loperamide; LTM: medium dose of lactitol (500 mg/kg) treated with loperamide; LTH: high dose of lactitol (800 mg/kg) treated with loperamide. ^#^ *p* < 0.05 and ^###^ *p* < 0.001 vs. NOR, and * *p* < 0.05 and ** *p* < 0.01 vs. CON.

Levels of serotonin, pivotal in orchestrating digestive tract peristalsis, and substance P, governing gastrointestinal tract contractions, intestinal motility, and gastric acid secretion, were quantified. Serotonin levels in CON were significantly lower compared to those in the NOR (*p* < 0.001). Lactitol administration resulted in a concentration-dependent increase in serotonin levels, with the LTH showing a significantly higher increase than the CON (Figure 5A, *p* < 0.05). Substance P content in the CON was significantly lower than that in the NOR (*p* < 0.05). Lactitol administration resulted in an increase in substance P in proportion to its concentration, and high-dose administration of lactitol resulted in a significantly higher substance P content than that in the CON (Figure 5B, *p* < 0.05). The observations indicate that lactitol administration contributed to an increase in the content of the intestinal neurotransmitters, serotonin and substance P, which contributed to increased peristalsis in the gastrointestinal tract.

### 3.6. Effect of Lactitol on Gene Expression Levels of Constipation-Related Genes

**Figure 6:**
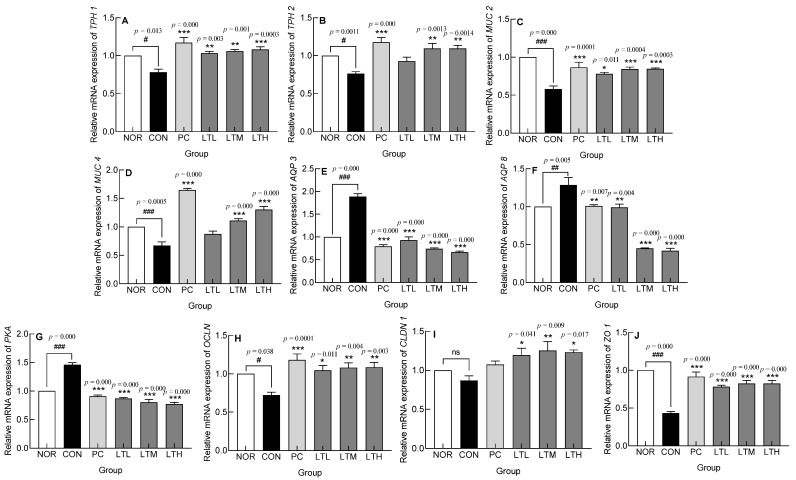
Effect of lactitol on gene expression levels of (**A**) Tryptophan hydroxylases (TPH)1, (**B**) TPH2, (**C**) Mucin (MUC)2, (**D**) MUC4, (**E**) Aquaporin (AQP)3, (**F**) AQP8, (**G**) Protein kinase A (PKA), (**H**) Occludin (OCLN), (**I**) Claudin (CLDN) and (**J**) Zonula occludens 1 (ZO1) in colon tissues of Sprague Dawley (SD) rats with loperamide-induced constipation. NOR: normal group; CON: loperamide-control group (5 mg/kg), PC: lactulose (2010 mg/kg) treated with loperamide; LTL: low dose of lactitol (300 mg/kg) treated with loperamide; LTM: medium dose of lactitol (500 mg/kg) treated with loperamide; LTH: high dose of lactitol (800 mg/kg) treated with loperamide. ^#^ *p* < 0.05, ^##^ *p* < 0.01 and ^###^ *p* < 0.001 vs. NOR, and * *p* < 0.05, ** *p* < 0.01 and *** *p* < 0.001 vs. CON in mRNA expression.

Gene expression was analyzed by RT-qPCR to evaluate the effect of lactitol administration on constipation (Figure 6). Expression of the serotonin synthesis enzymes, tryptophan hydroxylases (TPH1 and TPH2), showed that the CON had significantly lower levels than the NOR (Figure 6A,B, *p* < 0.05). TPH1 expression significantly increased in proportion with the treatment concentration in PC and lactitol-administered groups (Figure 6A, *p* < 0.001, *p* < 0.01, *p* < 0.01, and *p* < 0.001, respectively). The levels of TPH2 expression in the PC, LTM, and LTH were significantly higher than those in the CON (Figure 6B, *p* < 0.001, *p* < 0.01, and *p* < 0.01, respectively).

Mucin constitutes a vital element of the protective mucus layer covering intestinal mucosal cells, shielding the surface of intestinal epithelial cells against physical abrasion and chemical irritants. MUC2 expression was significantly lower in the CON than in the NOR (*p* < 0.001). Lactitol administration resulted in a significant increase in MUC2 expression in proportion with the dose as compared with that in the CON (Figure 6C, *p* < 0.05 and *p* < 0.001, respectively). With regards to MUC4, expression level in the CON was significantly lower than that in the NOR (*p* < 0.001). The administration of lactitol at medium and high doses (LTM and LTH) resulted in significantly higher MUC4 expression than that in the CON (Figure 6D, *p* < 0.001).

AQP is a membrane protein involved in intestinal water transport and plays an important role in the reabsorption of colonic fluids. Both AQP3 and 8 showed significantly higher expression in the CON than that in the NOR (Figure 6E,F, *p* < 0.001 and *p* < 0.01, respectively). The groups administered with lactitol (LTL, LTM, LTH) and lactulose (PC) demonstrated a notable reduction in expression levels relative to the CON, correlating with the dosage administered (Figure 6E,F). PKA, which promotes the transcription of the *AQP* gene through phosphorylation, showed a significant increase in expression in the CON compared to the NOR (Figure 6G, *p* < 0.001). PKA expression was significantly lower in the PC and lactitol treatment groups compared to the CON (Figure 6G).

Tight junctions contribute to the maintenance of the integrity of intestinal mucosal cells, which serve as the primary defense barrier. The expression of occludin (OCLN), claudin-1 (CLDN1), and ZO1, which are molecular structures that constitute tight junctions, were measured (Figure 6H–J). OCLN expression was significantly lower in the CON than that in the NOR (Figure 6H, *p* < 0.05). OCLN expression showed a significant dose-dependent increase compared to that in the CON (Figure 6H; *p* < 0.05, *p* < 0.01, and *p* < 0.01, respectively). CLDN1 expression in the CON was marginally reduced compared to the NOR, but this difference was not significant (Figure 6I). However, CLDN1 expression was significantly elevated in the LTL, LTM, and LTH compared to the CON. Though the highest expression was observed in the LTM, no significant difference was seen between the lactitol treatment groups (Figure 6I). The level of ZO1 expression was significantly lower in the CON than that in the NOR (Figure 6J, *p* < 0.001). In the lactitol administration groups (LTL, LTM, and LTH), ZO1 expression significantly increased in proportion with the concentration compared with that in the CON (Figure 6J, *p* < 0.001). Therefore, lactitol treatment showed an effect on suppressing the decrease in the expression of tight junction structural proteins OCLN, CLDN1, and ZO1 resulting from constipation.

### 3.7. Effect of Lactitol on c-kit, AQP3, and OCLN Protein Levels in Colonic Tissue of SD Rats with Loperamide-Induced Constipation

**Figure 7:**
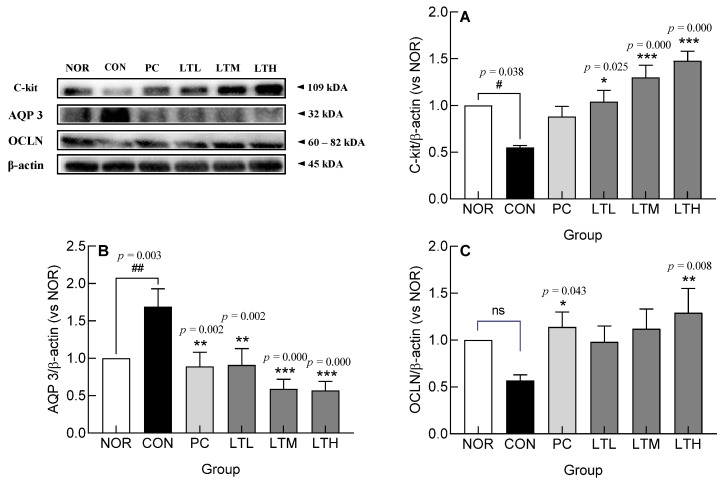
Effect of lactitol on protein levels of (**A**) c-kit, (**B**) Aquaporin 3 (AQP3) and (**C**) Occludin (OCLN) in colon tissues of SD rats treated with loperamide. NOR: normal group; CON: loperamide-control group (5 mg/kg), PC: lactulose (2010 mg/kg) treated with loperamide; LTL: low dose of lactitol (300 mg/kg) treated with loperamide; LTM: medium dose of lactitol (500 mg/kg) treated with loperamide; LTH: high dose of lactitol (800 mg/kg) treated with loperamide. ^#^ *p* < 0.05 and ^##^ *p* < 0.01 vs. NOR, and * *p* < 0.05, ** *p* < 0.01 and *** *p* < 0.001 vs. CON. ns: not significant.

Changes in the levels of c-kit, AQP3, and OCLN protein following lactitol administration were measured in loperamide-treated SD rats (Figure 7). The protein level of c-kit, a membrane receptor that generates the main signal for ICC activation, was significantly lower in the CON than that in the NOR (Figure 7A, *p* < 0.05). The c-kit protein level decreased following loperamide administration and was significantly higher than that in CON in proportion to the lactitol dose (Figure 7A, *p* < 0.05, *p* < 0.001, and *p* < 0.001, respectively). The expression of AQP3 protein, a crucial transporter of water in the large intestine, exhibited a marked elevation in the CON compared to the NOR (Figure 7B, *p* < 0.01). The AQP3 protein level decreased with constipation induction and was significantly lower than that in the CON in proportion with the lactitol-administered dose (Figure 7B, *p* < 0.01, *p* < 0.001, and *p* < 0.001, respectively). The level of OCLN protein, which is a tight junction molecular structure, tended to decrease in the CON compared with levels in the NOR; however, the difference was not significant (Figure 7C). The protein level of OCLN, which had been reduced due to the induction of constipation, tended to increase compared to that in the CON in proportion with the dose of lactitol administered. The levels of OCLN protein were significantly higher than those of CON only in the PC and LTH (Figure 7C, *p* < 0.05 and *p* < 0.01, respectively). Further, lactitol administration improved loperamide-induced changes in levels of c-kit, AQP3, and OCLN.

### 3.8. Effect of Lactitol on Short Chain Fatty Acids (SCFA) Content in Ceca of SD Rats with Loperamide-Induced Constipation

**Figure 8:**
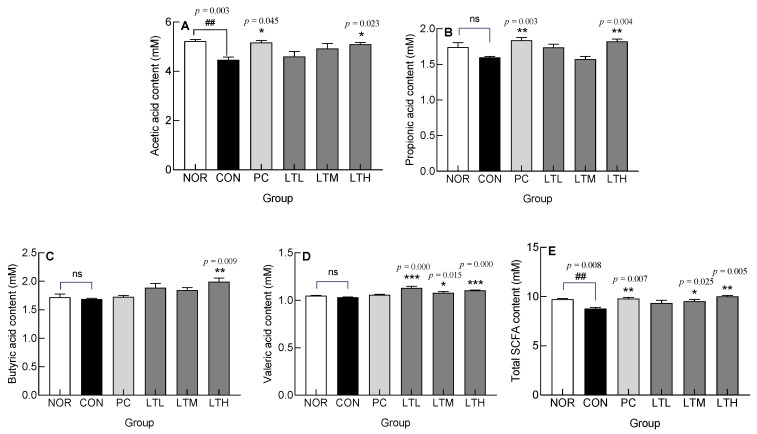
Effect of lactitol on content of (**A**) acetic acid, (**B**) propionic acid, (**C**) butyric acid, (**D**) valeric acid and (**E**) total short chain fatty acids (SCFA) in cecum of SD rats treated with loperamide. NOR: normal group; CON: loperamide-control group (5 mg/kg), PC: lactulose (2010 mg/kg) treated with loperamide; LTL: low dose of lactitol (300 mg/kg) treated with loperamide; LTM: medium dose of lactitol (500 mg/kg) treated with loperamide; LTH: high dose of lactitol (800 mg/kg) treated with loperamide. ^##^ *p* < 0.01 vs. NOR, and * *p* < 0.05, ** *p* < 0.01 and *** *p* < 0.001 vs. CON. ns: not significant.

To evaluate the effect of lactitol administration on loperamide-induced constipation in SD rats, the SCFA content in the cecum was measured by GC (Figure 8). Acetic, propionic, butyric, and valeric acid contents exhibited a tendency to be lower in the CON when compared to the NOR (Figure 8). The CON showed a significant decrease in acetic acid and total SCFA content compared with levels in the NOR (Figure 8A,E, *p* < 0.01, respectively). Among the total SCFA contents, the acetic acid content was the highest, and with an increase in the dose of lactitol, the acetic acid content tended to increase compared with that in the CON. A significantly greater level of acetic acid content than that in the CON was observed only with the administration of a high lactitol dose (Figure 8A, *p* < 0.05). Additionally, as compared with that in the CON, the LTM showed a significantly increased valeric acid content (Figure 8D, *p* < 0.05) along with a significantly higher total SCFA content (Figure 8E, *p* < 0.05). Likewise, the LTH had significantly increased SCFA components and total SCFA content compared with values in the CON (Figure 8). Although the administration of lactulose (PC) resulted in an increase in acetic, propionic, butyric, and valeric acid content compared to levels in the CON, only acetic and propionic acid content increased significantly (Figure 8A,B, *p* < 0.05 and *p* < 0.01, respectively). Figure 8E shows a notable increase in total SCFA levels in the PC compared to the CON, with statistical significance (*p* < 0.01). The SCFA content, which had decreased due to constipation, exhibited significant enhancements in the PC, LTM, and LTH.

### 3.9. Effects of Lactitol on Cecal Microbiota of SD Rats with Loperamide-Induced Constipation

Figure S1 depicts the changes in the species richness and diversity of the cecal microbiota induced by lactitol administration in loperamide-induced constipated SD rats. No significant difference was observed in the Chao1 index indicating species richness between the NOR and CON; however, the PC and LTH showed significantly lower Chao1 indices than that in the CON (Figure S1A, *p* < 0.05). The Shannon and Gini–Simpson indices, which represent species diversity, were significantly higher in the CON than that in NOR (*p* < 0.01 and *p* < 0.05, respectively). The three lactitol-treated groups had significantly lower Shannon and Gini–Simpson indices than that in the CON (Figure S1B,C, *p* < 0.01, *p* < 0.001, and *p* < 0.001, respectively).

To evaluate the differences in microbial community structure according to lactitol administration, beta-diversity results were visualized using PCoA based on the generalized UniFrac metric (Figure S2). The PCoA analysis with PC 2 and PC 3 as the axes revealed that the NOR was mainly located in the fourth quadrant, and the CON was mainly located in the second and third quadrants, showing differences in the distribution of the microbiota according to the separate locations between the groups (Figure S2). The PC was widespread in the third and fourth quadrants, confirming that it overlapped with the NOR and CON (Figure S2). When low doses of lactitol were administered, the microbiota were widely distributed in each quadrant; however, when high doses were administered, the microbiota were concentrated in the first quadrant. The distribution of microbiota in the LTH did not overlap with that in the CON and NOR.

**Figure 9:**
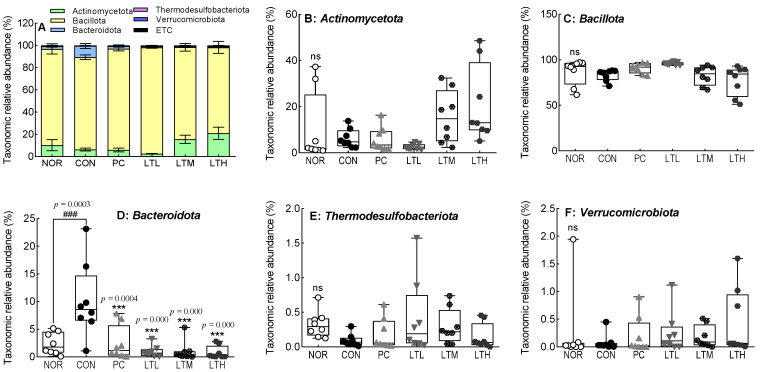
Effect of lactitol on the relative abundance of cecal microbiota at the phylum level in SD rats treated with loperamide. NOR, normal group, empty circle; CON, loperamide control group (5 mg/kg), filled circle; PC, lactulose (2010 mg/kg), filled triangle treated with loperamide; LTL, low-dose lactitol (300 mg/kg) treated with loperamide, upside down triangle; LTM, medium-dose lactitol (500 mg/kg) treated with loperamide, line hexagon; and LTH, high-dose lactitol (800 mg/kg), filled hexagon treated with loperamide. ^###^ *p* < 0.001 vs. NOR, and *** *p* < 0.001 vs. CON. ns: no significant.

The cecal microbiota at the phylum level showed that Bacillota (Firmicutes) were the most abundant, followed by Actinomycetota and Bacteroidota (Bacteroidetes) (Figure 9A). The relative abundance of Actinomycetota was slightly reduced when constipation was induced and slightly increased, though not significantly, with lactitol treatment (Figure 9B). In the CON, the relative abundance of Bacteroidota increased to a significantly higher level than that in the NOR. Figure 9D indicates a marked reduction in the relative abundance of Bacteroidota in the PC, LTL, LTM, and LTH compared to the CON with significance (*p* < 0.001).

**Figure 10:**
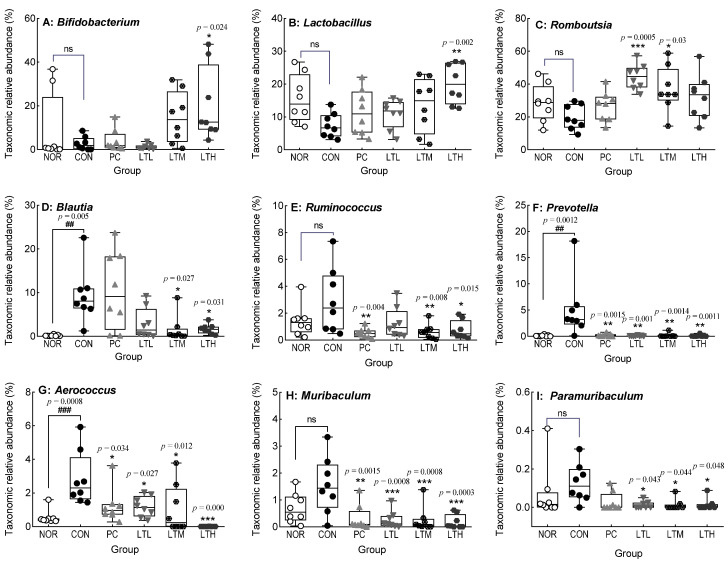
Effect of lactitol on the relative abundance of cecal microbiota at the genus level in SD rats treated with loperamide. NOR, normal group; CON, loperamide control group (5 mg/kg); PC, lactulose (2010 mg/kg) treated with loperamide; LTL, low-dose lactitol (300 mg/kg) treated with loperamide; LTM, medium-dose lactitol (500 mg/kg) treated with loperamide; and LTH, high-dose lactitol (800 mg/kg) treated with loperamide. ^##^ *p* < 0.01 and ^###^ *p* < 0.001 vs. NOR, and * *p* < 0.05, ** *p* < 0.01 and *** *p* < 0.001 vs. CON. ns: no significant.

Figure 10 shows the changes in the relative abundance of cecal microbiota at the genus level. The relative abundances of *Bifidobacterium*, *Lactobacillus*, and *Romboutsia* tended to decrease with the induction of constipation; however, no significant difference was observed between the NOR and CON (Figure 10A–C). The relative abundances of these strains tended to increase with the administration of lactulose and lactitol. The LTH showed a significantly increased abundance of *Bifidobacterium* and *Lactobacillus* compared with that in the CON (*p* < 0.05, *p* < 0.01, respectively). Similarly, the LTL and LTM showed an increased relative abundance of *Romboutsia* compared with that in the CON (*p* < 0.001 and *p* < 0.05, respectively). The relative abundances of *Blautia*, *Prevotella*, and *Aerococcus* in the CON were significantly higher than those in the NOR (Figure 10D,F,G; *p* < 0.01, respectively). The relative abundance of these strains significantly decreased in LTM and LTH compared with that in the CON. The relative abundances of *Ruminococcus*, *Muribaculum*, and *Paramuribaculum* showed a tendency to increase upon the induction of constipation; however, no significant difference was observed between the CON and NOR (Figure 10E,H,I). The relative abundances of *Muribaculum* and *Paramuribaculum* in the three lactitol groups were significantly reduced compared to the CON, corresponding to the lactitol dose (Figure 10H,I). The relative abundance of *Ruminococcus* also tended to decrease in proportion with the amount of lactitol administered, and the LTM and LTH showed significantly reduced relative abundances of this strain compared with that in the CON (Figure 10E, *p* < 0.01, and *p* < 0.05, respectively).

**Figure 11:**
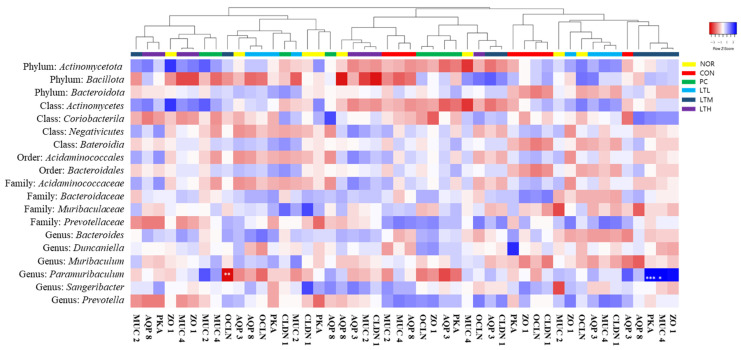
Pearson correlation analysis between intestinal barrier mobility factor and gut microbiota in SD rats treated with loperamide. NOR: normal group; CON: loperamide-control group (5 mg/kg), PC: lactulose (2010 mg/kg); LTL: low dose of lactitol treated group (300 mg/kg); LTM: medium dose of lactitol treated group (500 mg/kg); LTH: high dose of lactitol treated group (800 mg/kg). A deeper shade of blue indicates a stronger positive correlation, whereas a deeper shade of red signifies a stronger negative correlation. * *p* < 0.05, ** *p* < 0.01 and *** *p* < 0.001 by Pearson correlation.

Further, Pearson’s correlation analysis was performed to evaluate the correlation between the microbiome and biomarkers. This analysis between fecal parameters and the microbiome revealed no significant correlation (Figure S3). Pearson’s correlation analysis between constipation metabolites and the microbiome showed a significant positive correlation between the relative abundance of *Paramuribaculum* at the genus level and intestinal serotonin content in the LTM (Figure S4, *p* < 0.05). Pearson’s correlation analysis of the microbiome and intestinal barrier- and peristalsis-related biomarkers revealed a significant negative correlation in the LTM between the relative abundance of *Paramuribaculum* at the genus level and OCLN gene expression. The gene expression of PKA and MUC 4 and the relative abundance of *Paramuribaculum* showed significant positive correlations (Figure 11; *p* < 0.05, *p* < 0.01, and *p* < 0.001, respectively).

## 4. Discussion

Constipation is one of the most common digestive symptoms, with a reported prevalence as high as 20%. Lactitol, a hyperosmolar laxative, is a disaccharide derivative of galactose and sorbitol used to treat chronic functional constipation. This non-absorbed polysaccharide is fermented by intestinal microbiota and converted into fatty acids, resulting in increased osmosis and colonic motility.

The control of intestinal motility is achieved by the action of the visceral nervous system with different interactive functions [22], and colonic muscles are controlled by opposing mechanisms of relaxation and contraction [23]. Endogenous serotonin is secreted by the enterochromaffin cells in the epithelial cells of the mucosa in response to stimulation. In addition, the stimulation of 5-hydroxytryptamine receptor 4 present in primary afferent neurons inherent in the intestine induces peristalsis, thereby increasing stomach and intestinal movements and shortening transit time [24]. Substance P is an excitatory neurotransmitter that acts on neurokinin-1 receptor [25]. Increased serotonin and substance P levels enhance colonic motility. The increase in intestinal serotonin levels (Figure 5) appeared to be due to the increased expression of TPH1 and TPH2 (Figure 6A,B). Serotonin is synthesized by TpH-1 and TpH-2, which are present in enterochromaffin cells and serotonergic neurons in the myenteric plexus. Serotonin, which is secreted from the intestinal mucosa in response to stimuli, such as the inflow of food, binds to receptors in the splanchnic plexus and plays an important role in regulating the sensory and motor functions of the gastrointestinal tract [26]. The findings of this study indicate that the lactitol-mediated increase in serotonin and substance P levels (Figure 5) led to increased intestinal motility and transit rate (Figure 2). Serotonin content increased with medium doses of lactitol (LTM) and was positively correlated with the relative abundance of *Paramuribaculum* (Figure S4). AQP, which is responsible for water transport within the gastrointestinal tract, is also involved in fluid transport because its transcription is promoted by PKA phosphorylation. AQP3 and AQP8 play major roles in water reabsorption by the colonic mucosal epithelial cells [27,28]. PKA, AQP3, and AQP8 expression was increased by constipation induction; however, lactitol treatment decreased their expression in a concentration-dependent manner (Figure 6E–G). The AQP3 protein level also decreased following lactitol administration (Figure 7B). The lactitol-mediated downregulation of PKA, AQP3, and AQP8 mRNA and/or protein levels was seen to contribute to an increase in the water content of fecal pellets (Figure 1C). In particular, the increase in PKA mRNA levels in the LTM positively correlated with the relative abundance of *Paramuribaculum* (Figure 11, *p* < 0.01).

To prevent epithelial cells from being damaged by chemical stimulation or friction in the colon, mucus, the main component of which is mucin, is secreted to protect the colonic contents or participate in their movement [29]. In constipation, mucus secretion in the intestinal mucosa is reduced, thereby inhibiting the movement of the colonic contents. Loperamide reduces colonic motility, along with mucus secretion and crypt cell distribution in the colon [30], as observed in H&E and Alcian staining (Figure 1). Lactitol administration upregulates the expression levels of MUC2 [31], which is synthesized in goblet cells and is mostly secreted in the colon, and MUC4 is a membrane-bound mucin [29] that increases the mucosal layer thickness and gastrointestinal transit rate [32]. Furthermore, the elevation in MUC4 gene expression exhibited a significant positive correlation with *Paramuribaculum* following the administration of a medium dose of lactitol (Figure 11, *p* < 0.05). The administration of 300 and 500 mg/kg lactitol improved constipation by increasing the mucus layer and the distribution of crypt cells (Figure 3A and Figure 4B). Further, levels of ICC [33], which are distributed in the intestines and known to soften the intestines and enable effective intestinal peristalsis, were also increased by lactitol administration (Figure 4B). The increased distribution of ICC is closely related to c-kit., and ICC differentiation, maturation, and function are regulated by the c-kit/SCF signaling pathway [34]. As the concentration of lactitol increased, the c-kit protein levels also increased (Figure 7A). The increase in c-kit protein levels contributed to an increase in ICC, which appeared to have contributed to the increase in colonic motility.

The human intestine is a reservoir of intestinal microbiota and has a series of defense systems, including mucosal barrier function, immunoglobulin secretion function, and the macrophage system [35]. The intestinal mucosa has a dual function as a barrier that blocks external substances while simultaneously allowing them to pass through and be absorbed. The barrier function is regulated by three complexes: adherent junctions, desmosomes, and tight junctions [36]. Tight junctions are protein complexes containing ZO-1, CLDN1, and OCLN, which act as intestinal barriers by sealing adjacent epithelial cells [37,38]. We noted that the expression of tight junction proteins was decreased by loperamide treatment and restored to above normal levels by lactitol administration (Figure 6H–J). Lactitol administration restored OCLN to levels above those in the NOR (Figure 7C). The intestinal barrier function, which was reduced due to constipation, was improved by lactitol administration. The level of OCLN expression in LTM showed a significant positive correlation with intestinal *Paramuribaculum* (Figure 11, *p* < 0.01).

Emerging evidence suggests that the composition of intestinal microorganisms differs between patients with functional constipation. As a prebiotic, lactitol produces SCFAs, which are the main energy sources of intestinal microorganisms; promotes the growth of beneficial intestinal bacteria; and improves constipation by promoting intestinal peristalsis [39,40]. Lactitol administration increases the total SCFA and butyric acid contents [41,42]. An increase in butyric acid levels has been confirmed in cecal fermentation in vitro [43]. These findings support our current observations of elevated levels of both butyric acid and total SCFA contents following the administration of a high dose of lactitol to SD rats (Figure 8C,E). The relative abundance of *Firmicutes* and *Bacteroidetes*, the dominant bacteria in the feces of humans and mice, respectively [44], was decreased and increased by inducing constipation [45]. The administration of lactitol resulted in a significant reduction in the relative abundance of Bacteroidota (Bacteroidetes), which had previously increased due to constipation (Figure 9D). This suggests a mitigated effect on constipation. Conversely, lactitol administration increased the relative abundance of *Bifidobacterium*, *Lactobacillus*, and *Romboutsia*, which are all beneficial at the genus level (Figure 10A–C). The lactitol-mediated increase in *Bifidobacterium* and *Lactobacillus* seems to be associated with intestinal acidification and a reduction in ammonia production and absorption, which in turn increases the number of beneficial bacteria and selectively reduces putrefactive bacteria [46,47]. *Romboutsia* contributes to intestinal motility and the production of SCFA [48,49], which play important physiological roles in the host [50]. The increased abundance of *Lactobacillus* and *Romboutsia* also contributes to improvements in fecal parameters [51]. The increase in *Lachnospiraceae* at the family level was due to an increase in *Blautia* and *Ruminococcus* at the genus level [52]. The increase in *Prevotella, Aerococcus* [53], and *Muribaculum* [54] in constipation-induced rats was significantly decreased by lactitol administration (Figure 10D–H). In a constipation-induced aging model in SD rats following excessive galactose and loperamide administration, the level of *Prevotella* increased [55], which is consistent with our results (Figure 10F). However, as the level of *Muribaculum* significantly decreased [56], which is in contrast to our results, further research is needed to clarify the relationship of this genus with constipation. Valeric acid was shown to be negatively and positively correlated with *Muribaculum* and *Lactobacillus*, respectively [57]. Compared to values in the constipation-induced model, the lactitol administration group showed an increase in valeric acid content (Figure 8D) and changes in the relative abundance of *Lactobacillus* and *Muribaculum* (Figure 10B,H), the findings being similar to those of a recent study [57].

The administration of lactitol has demonstrated efficacy in alleviating constipation through several physiological mechanisms. First, lactitol enhances the expression of serotonin synthesis enzymes TPH1 and TPH2, thereby promoting increased serotonin production, which in turn enhances gastrointestinal motility. Additionally, lactitol increases the expression of mucin genes, such as MUC2 and MUC4, which play pivotal roles in maintaining the integrity of the intestinal mucosal barrier. Moreover, lactitol upregulates tight junction proteins including OCLN, CLDN1, and ZO-1, which strengthen the intestinal barrier. Furthermore, lactitol downregulates water and electrolyte regulators such as AQP3, AQP8, and PKA, contributing to improved stool consistency. Lastly, lactitol promotes the growth of beneficial gut bacteria, such as *Bifidobacterium* and *Lactobacillus* and increases SCFA levels, thereby optimizing the intestinal environment. Collectively, these regulations contribute to improvements in constipation. Lactitol is used as a food ingredient and provides better safety and taste than lactulose. However, to progress clinical application, the safety of lactitol must be evaluated through acute and subacute dosing studies. Additionally, further investigation is required to elucidate the mechanisms by which cecal microbiota interact with constipation-related factors.

## 5. Conclusions

The current study confirmed that lactitol has the ability to alleviate constipation by regulating constipation-related biomarkers in intestinal microorganisms. Consequently, lactitol has emerged as a promising food-grade laxative with the potential to replace lactulose, a *hyperosmotic laxative* used to treat constipation.

## Data Availability

The original contributions presented in the study are included in the article and Supplementary Materials, further inquiries can be directed to the corresponding author.

## Supplementary Materials

The following supporting information can be downloaded at: https://www.mdpi.com/article/10.3390/foods13132128/s1, Figure S1. Effect of lactitol on (A) Chao1, (B) Shannon, and (C) Gini–Simpson indices in the ceca of SD rats treated with loperamide. Figure S2. Principal coordinate analysis of microbial community composition by lactitol treatment in SD rats treated with loperamide. Figure S3. Pearson correlation analysis between fecal parameters and gut microbiota in SD rats treated with loperamide. Figure S4. Pearson correlation analysis between constipation metabolites and gut microbiota in SD rats treated with loperamide. Table S1. Oligonucleotide primers used for polymerase chain reaction (PCR).
