# Supplementary material for: Lactitol Alleviates Loperamide-Induced Constipation in Sprague Dawley Rats by Regulating Serotonin, Short-Chain Fatty Acids, and Gut Microbiota
Source: Foods. 2024 Jul 3;13(13):2128. doi: 10.3390/foods13132128 (PMC11240941; doi:10.3390/foods13132128)
Supplement: Supplementary file 1 [file foods-13-02128-s001.zip › foods-3048259-Figure S1.pdf]

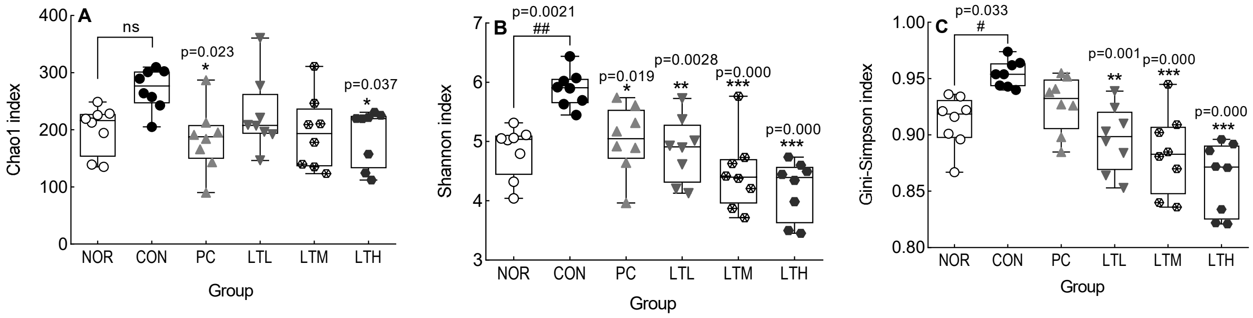

Figure S1. Effect of lactitol on (A) Chao1, (B) Shannon and (C) Gini-Simpson indices in the ceca of SD rats treated with loperamide. NOR: normal group; CON: loperamide-control group (5 mg/kg), PC: lactulose (2010 mg/kg) treated with loperamide; LTL: low dose of lactitol (300 mg/kg) treated with loperamide; LTM: medium dose of lactitol (500 mg/kg) treated with loperamide; LTH: high dose of lactitol (800 mg/kg) treated with loperamide. Data are expressed means ± standard error of the mean (S.E.M) (n=6). $^{\#}p < 0.05$ and $^{\#\#}p < 0.01$ vs. NOR, and $^{*}p < 0.05$, $^{**}p < 0.01$ and $^{***}p < 0.001$ vs. CON by Tukey's test.
