# Supplementary material for: Lactitol Alleviates Loperamide-Induced Constipation in Sprague Dawley Rats by Regulating Serotonin, Short-Chain Fatty Acids, and Gut Microbiota
Source: Foods. 2024 Jul 3;13(13):2128. doi: 10.3390/foods13132128 (PMC11240941; doi:10.3390/foods13132128)
Supplement: Supplementary file 1 [file foods-13-02128-s001.zip › foods-3048259-Figure S2.pdf]

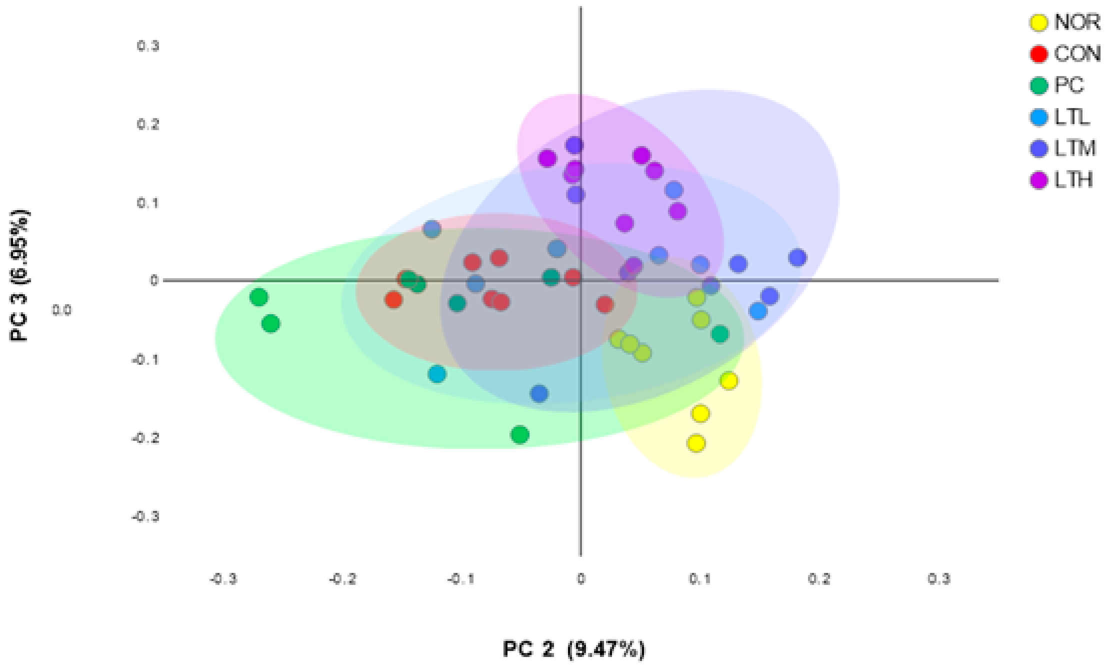

Figure S2. Principal coordinate analysis of microbial community composition by lactitol treatment in SD rats treated with loperamide. NOR: normal group; CON: loperamide-control group (5 mg/kg); PC: lactulose (2010 mg/kg); LTL: low-dose lactitol-treated group (300 mg/kg); LTM: medium-dose lactitol-treated group (500 mg/kg); LTH: high-dose lactitol-treated group (800 mg/kg).
