# Supplementary material for: Lactitol Alleviates Loperamide-Induced Constipation in Sprague Dawley Rats by Regulating Serotonin, Short-Chain Fatty Acids, and Gut Microbiota
Source: Foods. 2024 Jul 3;13(13):2128. doi: 10.3390/foods13132128 (PMC11240941; doi:10.3390/foods13132128)
Supplement: Supplementary file 1 [file foods-13-02128-s001.zip › foods-3048259-Table S1.pdf]

Table S1. Oligonucleotide primers used for polymerase chain reaction (PCR)

Target gene		PCR primer sequence (5' to 3')	Target gene		PCR primer sequence (5' to 3')
SERT	F	ATCTCCTAGAACCCTGTAAC	TPH 1	F	CACTCACTGTCTCTGAAAACGC
	R	GAAATGGACCTGGAGTATTG		R	AGCCATGAATTTGAGAGGGAGG
TPH 2	F	TAAATACTGGGCCAGGAGAGG	AQP3	F	CACTTGGATATGGTCAATGGCTC
	R	GAAGTGTCTTTGCCGCTTCTC		R	GGGTTGTTATAAGGGTCAACAATGG
AQP 8	F	GGCCTCAAGACCATGCTGCTA	PKA	F	CAGGAAAGCGCTCCAGATAC
	R	ACCTGCTCCTGCTCCTGGACTA		R	AAGGGAAGGTTGGCGTTACT
MUC 2	F	CAAGTGATTGTGTTTCAGGCTC	MUC 4	F	GACAAAGCACCAATTCCATCC
	R	TGGAGATGTTCTTGGTGCAG		R	CCTTAGAGTTGCTGGTGATCT
ZO1	F	AAAAGTGAACCACGAGATGCT	OCLN	F	CACACAGGACATGCCTCCAC
	R	AAAGCTGGAGGACTGGAGATGA		R	GGCTGCCTGAAGTCATCCAC
CLDN 1	F	ACGAGACCGTCAAGGCCAAG	β-actin	F	ACCGTGAAAAGATGACCCAGAT
	R	GTCCAGGACACAGGCACCATAA		R	CCAGAGGCATACAGGGACAA

SERT, serotonin reuptake transporter; TPH 1, tryptophan hydroxylase 1; TPH 2, tryptophan hydroxylase 2; AQP 3, aquaporin 3; AQP 8, aquaporin 8; PKA, protein kinase A; MUC 2, mucin 2; MUC 4, mucin 4; ZO-1, zonula occludens 1; OCLN, occludin; CLDN-1, claudin 1.
